# QuickStats

**Published:** 2015-07-31

**Authors:** FROM THE NATIONAL CENTER FOR HEALTH STATISTICS

**Figure:**
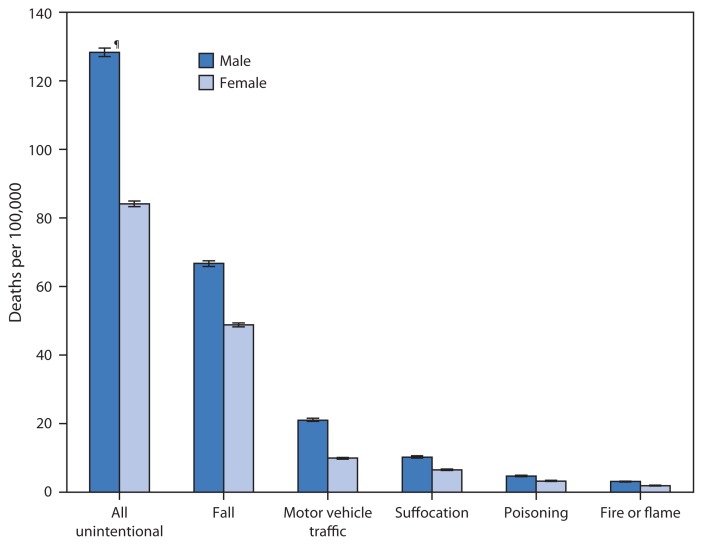
Death Rates* from Unintentional Injury Among Adults Aged ≥65 Years, by Cause of Death^†^ and Sex — National Vital Statistics System, United States, 2012–2013 * Per 100,000 population, age-adjusted to the 2000 U.S. standard population. ^†^ Unintentional injury deaths are identified using the *International Classification of Diseases, Tenth Revision* (ICD-10) underlying cause of death codes V01-X59, Y85-Y86 (all unintentional); W00-W19 (fall); [V02–V04](.1,.9), V09.2, [V12–V14](.3–.9), V19(.4–.6), [V20–V28](.3–.9), [V29–V79](.4–.9), V80(.3–.5), V81.1, V82.1, [V83–V86](.0–.3), V87(.0–.8), V89.2 (motor vehicle traffic); W75-W84 (suffocation); X40-X49 (poisoning); and X00-X09 (fire or flame). ^¶^ 95% confidence interval.

During 2012–2013, among persons aged ≥65 years, men had higher age-adjusted death rates than women from all unintentional injuries, (128.3 versus 84.1 deaths per 100,000 population, respectively), and from the five leading causes of unintentional injury death. Death rates due to falls were the highest for both men and women, with the rates for men 1.4 times higher than the rates for women (66.7 versus 48.8). Compared to the age-adjusted death rates for women, the rates for men were 2.1 times higher for motor vehicle traffic crashes (21.0 versus 9.9).

**Source:** National Vital Statistics System mortality data. Available at http://www.cdc.gov/nchs/deaths.htm.

**Reported by:** Ellen A. Kramarow, PhD, ekramarow@cdc.gov, 301-458-4325; Yahtyng Sheu, PhD.

